# Causes of death after emergency general surgical admission: population cohort study of mortality

**DOI:** 10.1093/bjsopen/zrab021

**Published:** 2021-04-21

**Authors:** G Ramsay, J M Wohlgemut, M Bekheit, A J M Watson, J O Jansen

**Affiliations:** Department of General Surgery, Aberdeen Royal Infirmary, Aberdeen, UK; Rowett Institute for Health, Foresterhill, University of Aberdeen, Aberdeen, UK; Department of General Surgery, Queen Elizabeth University Hospital, Glasgow, UK; Department of General Surgery, Aberdeen Royal Infirmary, Aberdeen, UK; Department of Surgery, Elkabbary Hospital, Alexandria, Egypt; Department of Surgery, Raigmore Hospital, Inverness, UK; Division of Acute Care Surgery, Department of Surgery, University of Alabama at Birmingham, Birmingham, Alabama, USA

## Abstract

**Background:**

A substantial number of patients treated in emergency general surgery (EGS) services die within a year of discharge. The aim of this study was to analyse causes of death and their relationship to discharge diagnoses, in patients who died within 1 year of discharge from an EGS service in Scotland.

**Methods:**

This was a population cohort study of all patients with an EGS admission in Scotland, UK, in the year before death. Patients admitted to EGS services between January 2008 and December 2017 were included. Data regarding patient admissions were obtained from the Information Services Division in Scotland, and cross-referenced to death certificate data, obtained from the National Records of Scotland.

**Results:**

Of 507 308 patients admitted to EGS services, 7917 died while in hospital, and 52 094 within 1 year of discharge. For the latter, the median survival time was 67 (i.q.r. 21–168) days after EGS discharge. Malignancy accounted for 48 per cent of deaths and was the predominant cause of death in patients aged over 35 years. The cause of death was directly related to the discharge diagnosis in 56.5 per cent of patients. Symptom-based discharge diagnoses were often associated with a malignancy not diagnosed on admission.

**Conclusion:**

When analysed by subsequent cause of death, EGS is a cancer-based specialty. Adequate follow-up and close links with oncology and palliative care services merit development.

## Teaser

Emergency general surgery is less well studied than other aspects of the general surgery specialty. The authors have previously documented a strikingly high postdischarge mortality rate; causes of death in this cohort are assessed here.

## Introduction

Emergency general surgery (EGS) comprises the unscheduled in-hospital treatment of patients under the care of a surgeon with training in gastrointestinal surgery^1–6^, and an important part of the spectrum of care provided by general surgeons^7^^,^^8^. At any one time, around half of general surgical beds in the UK are occupied by patients who were admitted as an emergency^1^. Per annum, there are around 74 000 such admissions in Scotland^4^ and approximately 3 million in the USA^9^^,^^10^.

Patients in the EGS service, regardless of operative intervention, are at high risk of dying. The in-hospital mortality rate for such patients is approximately eight times that of patients admitted for elective general surgery^11^. The mortality rate remains high after discharge, particularly among older patients, and those with co-morbidities^5^. In Scotland, 35 per cent of patients aged 75 years and older, who were admitted as an emergency under the care of a general surgeon, died within 1 year of discharge, a figure that almost doubled when severe co-morbidities were present^3^.

Studies related to EGS have often concentrated on patients who have undergone operations, especially laparotomy^2^^,^^7^^,^^12–14^, although only a small proportion require operative treatment^5^^,^^15^. Conservatively managed patients are a large group, and these patients also have a high mortality risk, about double that of patients having surgery^3^.

Although previous analyses have highlighted poor long-term outcomes after EGS admission and demonstrated the significant influence of age and co-morbidity^3^, causes of postdischarge mortality remain unclear. It is not known whether these patients die from conditions related to the EGS admission, or from entirely different causes. This has obvious implications for postdischarge care and follow-up. The aim of the present study was to analyse causes of death and their relationship with discharge diagnoses, in patients who died within 1 year of discharge from an EGS service in Scotland.

## Methods

This was a population‐based, cross‐sectional study in Scotland, UK.

### Data sources

The Information Services Division (ISD; https://www.isdscotland.org) of National Health Service (NHS) Scotland records data on all interactions with the NHS. Patients are assigned a unique identifier (Community Health Index (CHI) number^16^) on first contact with NHS Scotland services, which allows healthcare interactions to be tracked over time, regardless of provider. ISD data are linked to national death records, allowing mortality to be examined, irrespective of death as an inpatient or in the community.

The ISD uses a consistent coding strategy and data are abstracted by professional coders, with high accuracy and consistency^17^. Data are coded locally within each health board, and stored centrally. Diagnoses are coded using ICD‐10, and operative codes using OPCS-4. For the purpose of this study, individualized data for patients meeting the inclusion criteria were anonymized at source and transferred to the National Data Safehaven for analysis. Demographic details, diagnoses, dates of admission, co-morbidity status (according to the Charlson Co-morbidity Index (CCI), 10-year look back)^18^, and discharge information were obtained from the SMR01 national data set. Date and cause of death (also coded by ICD-10)^19^ were obtained from the National Records of Scotland. For comparison of discharge diagnoses and causes of death, both primary and secondary causes of death were included. Causes of death for the population of Scotland as a whole were obtained from the National Register of Scotland^20^.

### Patient cohort

The study included patients aged 16 years and older, who had an unplanned non-elective (emergency) admission to hospital under the care of a consultant general surgeon (specialty code C11), between January 2008 and December 2017. Unscheduled transfers into this service from another hospital ward or hospital were also included. Postadmission healthcare interactions and death (either as an inpatient or in the community) were tracked by linking records via the CHI number until the date of death or December 2018.

### Permissions

The project was approved by the Public Benefit and Privacy Panel of NHS Scotland (reference 1819‐0340) and registered with the research governance department of NHS Grampian and the University of Aberdeen.

### Statistical analysis

The relationship between discharge diagnosis and cause of death was analysed descriptively, using colour-coded matrices containing the 50 most common primary discharge diagnoses and causes of death. The study population was analysed as a whole, as well as for patients who underwent operative treatment during the last admission and those managed without surgery.

Data were analysed using Microsoft Excel^®^ version 16.0 (Microsoft, Redmond, Washington, USA) and SPSS^®^ version 24.0 (IBM, Armonk, New York, USA). Categorical data were analysed with χ^2^ tests, and ordinal data using Mann–Whitney *U* tests. Graphs were created using DataGraph (Visual Data Tools, Chapel Hill, NC, USA).

## Results

A total of 507 308 patients were identified, resulting in 814 790 admission episodes over the 10 years of study. Of these, 7917 patients died in hospital and 499 391 were discharged; 52 094 patients (10.4 per cent) died within 1 year of discharge. The median age at time of death was 76 (i.q.r. 66–84) years. Some 1.2 per cent of patients who died within 1 year were aged 34 years or younger, 9.0 per cent were aged 35–54 years, 36.5 per cent aged 55–74 years, and 53.3 per cent aged 75 years or older. The majority of patients had moderate (CCI score 1–4, 50.2 per cent) or severe (CCI score over 4, 38.8 per cent) co-morbidities. Only 10.9 per cent of patients who died had no co-morbidities. The median interval between discharge and death was 67 (i.q.r. 21–168) days. A total of 13 700 patients (26.3 per cent) had an operation during their last admission. Those who had an operation died a median of 64 (19–164) days after discharge and those managed without surgery died 68 (21–169) days after discharge.

### Postdischarge causes of death

*Tables 1* and *2* show causes of death for 13 700 patients who had an operation and 38 394 treated without surgery respectively. Overall, malignancies accounted for almost half of all deaths: 7280 (53.1 per cent) in the operative cohort and 17 735 (46.2 per cent) in the non-operative cohort (*P* < 0.001). Among patients who had surgery, cancer of the oesophagus was the most common cause of death, followed by cancers of the colon, pancreas, bronchus or lung, and stomach. The most common non-malignant causes of death were acute myocardial infarction (rank 7), chronic ischaemic heart disease, and chronic obstructive pulmonary disease (COPD). In patients who did not require an operation, colonic cancer was the most common cause of death, followed by malignancies in the bronchus or lung, pancreas, and oesophagus. Chronic ischaemic heart disease (rank 5), COPD, and acute myocardial infarction were the most common non-neoplastic causes of death.

**Table 1:** Most common discharge diagnoses and causes of death for 13 700 patients who died within 1 year of emergency general surgery admission, and had operative treatment during the last admission

Causes of death				Discharge diagnoses			
Rank	ICD-10 code	Description	*n*	Rank	ICD-10 code	Description	*n*
1	C15	Malignant neoplasm of oesophagus	1040 (7.6)	1	C18	Malignant neoplasm of colon	944 (6.9)
2	C18	Malignant neoplasm of colon	952 (6.9)	2	K56	Paralytic ileus and intestinal obstruction without hernia	929 (6.8)
3	C25	Malignant neoplasm of pancreas	820 (6.0)	3	C15	Malignant neoplasm of oesophagus	766 (5.6)
4	C34	Malignant neoplasm of unspecified part of bronchus or lung	470 (3.4)	4	C25	Malignant neoplasm of pancreas	571 (4.2)
5	C16	Malignant neoplasm of stomach	463 (3.4)	5	C78	Secondary malignant neoplasm of respiratory and digestive organs	385 (2.8)
6	C22	Malignant neoplasm of liver and intrahepatic bile ducts	385 (2.8)	6	C16	Malignant neoplasm of stomach	358 (2.6)
7	I21	Acute myocardial infarction	376 (2.7)	7	K57	Diverticular disease of intestine	349 (2.6)
8	I25	Chronic ischaemic heart disease	362 (2.6)	8	K80	Cholelithiasis	334 (2.4)
9	C80	Malignant neoplasm without specification of site	318 (2.3)	9	K55	Vascular disorders of intestine	300 (2.2)
10	J44	Chronic obstructive pulmonary disease	318 (2.3)	10	R33	Retention of urine	275 (2.0)
11	C20	Malignant neoplasm of rectum	297 (2.2)	11	T85	Complications of other internal prosthetic devices, implants, and grafts	272 (2.0)
12	C19	Malignant neoplasm of rectosigmoid junction	292 (2.1)	12	C20	Malignant neoplasm of rectum	271 (2.0)
13	J18	Pneumonia, unspecified organism	269 (2.0)	13	K92	Haematemesis/melaena	250 (1.8)
14	K56	Paralytic ileus and intestinal obstruction without hernia	255(1.9)	14	C22	Malignant neoplasm of liver and intrahepatic bile ducts	234 (1.7)
15	K55	Vascular disorders of intestine	228 (1.7)	15	K62	Other diseases of anus and rectum	234 (1.7)
16	C26	Malignant neoplasm of other and ill defined digestive organs	220 (1.6)	16	K63	Other diseases of intestine (including perforation)	226 (1.7)
17	C56	Malignant neoplasm of ovary	220 (1.6)	17	R10	Abdominal and pelvic pain	216 (1.6)
18	C61	Malignant neoplasm of prostate	189 (1.4)	18	L02	Cutaneous abscess, furuncle, and carbuncle	205 (1.5)
19	C50	Malignant neoplasm of breast	187 (1.4)	19	K40	Inguinal hernia	197 (1.4)
20	C67	Malignant neoplasm of bladder	179 (1.3)	20	K26	Duodenal ulcer	192 (1.4)
21	K57	Diverticular disease of intestine	156 (1.1)	21	C80	Malignant neoplasm without specification of site	188 (1.4)
22	I64	Stroke, unspecified	155 (1.1)	22	K22	Other diseases of oesophagus	188 (1.4)
23	K70	Alcoholic liver disease	155 (1.1)	23	K59	Constipation	184 (1.3)
24	J69	Pneumonitis due to solids and liquids	145 (1.1)	24	K83	Other diseases of biliary tract	182 (1.3)
25	K63	Other diseases of intestine (including perforation)	133 (1.0)	25	R13	Aphagia and dysphagia	152 (1.1)

Values in parentheses are percentages.

**Table 2:** Most common discharge diagnoses and causes of death for 38 394 patients who died within 1 year of emergency general surgery admission, and had non-operative treatment during the last admission

Causes of death				Discharge diagnoses			
Rank	ICD-10 code	Description	*n*	Rank	ICD-10 code	Description	*n*
1	C18	Malignant neoplasm of colon	1931 (5.0)	1	R10	Abdominal and pelvic pain	6397 (16.7)
2	C34	Malignant neoplasm of unspecified part of bronchus or lung	1824 (4.8)	2	K59	Constipation	1912 (5.0)
3	C25	Malignant neoplasm of pancreas	1812 (4.7)	3	K56	Paralytic ileus and intestinal obstruction without hernia	1312 (3.4)
4	C15	Malignant neoplasm of oesophagus	1309 (3.4)	4	C18	Malignant neoplasm of colon	1164 (3.0)
5	I25	Chronic ischaemic heart disease	1255 (3.3)	5	C25	Malignant neoplasm of pancreas	1161 (3.0)
6	J44	Chronic obstructive pulmonary disease	1210 (3.2)	6	K92	Haematemesis	1104 (2.9)
7	I21	Acute myocardial infarction	1166 (3.0)	7	N39	Urinary tract infection, site not specified	906 (2.4)
8	C22	Malignant neoplasm of liver and intrahepatic bile ducts	959 (2.5)	8	K85	Acute pancreatitis	854 (2.2)
9	J18	Pneumonia, unspecified organism	946 (2.5)	9	K80	Cholelithiasis	826 (2.2)
10	C50	Malignant neoplasm of breast	827 (2.2)	10	C15	Malignant neoplasm of oesophagus	774 (2.0)
11	C16	Malignant neoplasm of stomach	816 (2.1)	11	K62	Other diseases of anus and rectum	750 (2.0)
12	C61	Malignant neoplasm of prostate	789 (2.1)	12	S09	Other and unspecified injuries of head	719 (1.9)
13	C67	Malignant neoplasm of bladder	723 (1.9)	13	K57	Diverticular disease of intestine	684 (1.8)
14	C20	Malignant neoplasm of rectum	697 (1.8)	14	C78	Secondary malignant neoplasm of respiratory and digestive organs	679 (1.8)
15	C80	Malignant neoplasm without specification of site	691 (1.8)	15	A41	Sepsis	663 (1.7)
16	C19	Malignant neoplasm of rectosigmoid junction	612 (1.6)	16	C34	Malignant neoplasm of bronchus and lung	515 (1.3)
17	C56	Malignant neoplasm of ovary	574 (1.5)	17	R11	Nausea and vomiting	505 (1.3)
18	F03	Unspecified dementia	554 (1.4)	18	C16	Malignant neoplasm of stomach	489 (1.3)
19	K70	Alcoholic liver disease	637 (1.4)	19	I73	Other peripheral vascular diseases	481 (1.3)
20	C26	Malignant neoplasm of other and ill defined digestive organs	490 (1.3)	20	S01	Open wound of head	480 (1.3)
21	I69	Sequelae of cerebrovascular disease	468 (1.2)	21	C22	Malignant neoplasm of liver and intrahepatic bile ducts	460 (1.2)
22	I73	Other peripheral vascular diseases	454 (1.2)	22	J18	Pneumonia, unspecified organism	442 (1.2)
23	F01	Vascular dementia	463 (1.2)	23	K83	Complications of genitourinary prosthetic devices, implants, and grafts	416 (1.1)
24	K56	Paralytic ileus and intestinal obstruction without hernia	441 (1.2)	24	T81	Complications of procedures, not elsewhere classified	396 (1.0)
25	R68	Other general symptoms and signs (including hypothermia)	428 (1.1)	25	K55	Vascular disorders of intestine	385 (1.0)

Values in parentheses are percentages.

The 50 most common causes of death (which accounted for 76.6 per cent of all deaths) for the 52 094 patients who died within 1 year of EGS admission are detailed in *Table S1*.

The ranking of causes of death varied with age (*Table 3*). Of the 619 patients who died aged 16–34 years, poisoning and substance abuse (18.3 per cent), alcoholic liver disease (6.1 per cent), and deliberate self-harm (4.4 per cent) were the most common causes. In those aged 35–54 years (4670 deaths), alcoholic liver disease (7.1 per cent) was the most common cause, followed by colonic, breast, pancreatic, oesophageal, and lung cancers. In 55–74 year olds, neoplastic conditions continued to predominate, whereas among patients aged 75 years and older, acute myocardial infarction, pneumonia, and COPD began to feature more heavily.

**Table 3:** Most common cause of death by age group for patients who died within 1 year of emergency general surgery admission

Rank	ICD-10 code	Description	*n*
**Age 16–34 years**			
1	X42	Accidental poisoning by and exposure to narcotics and psychodysleptics	62 (10.0)
2	K70	Alcoholic liver disease	38 (6.1)
3	F19	Other psychoactive substance dependence with intoxication with perceptual disturbance	32 (5.2)
4	X70	Intentional self harm	27 (4.4)
5	C18	Malignant neoplasm of colon	22 (3.6)
6	C50	Malignant neoplasm of breast	19 (3.1)
7	Y12	Poisoning by and exposure to narcotics and psychodysleptics	19 (3.1)
8	C53	Malignant neoplasm of cervix	16 (2.6)
9	K85	Acute pancreatitis	13 (2.1)
10	C20	Malignant neoplasm of rectum	12 (1.9)
All deaths			619
**Age 35–54 years**			
1	K70	Alcoholic liver disease	333 (7.1)
2	C18	Malignant neoplasm of colon	246 (5.3)
3	C50	Malignant neoplasm of breast	242 (5.2)
4	C25	Malignant neoplasm of pancreas	230 (4.9)
5	C15	Malignant neoplasm of oesophagus	218 (4.7)
6	C34	Malignant neoplasm of unspecified part of bronchus or lung	155 (3.3)
7	X42	Accidental poisoning by and exposure to narcotics and psychodysleptics	119 (2.5)
8	C16	Malignant neoplasm of stomach	115 (2.5)
9	F10	Alcohol abuse	103 (2.2)
10	C19	Malignant neoplasm of rectum	100 (2.1)
All deaths			4670
**Age 55–74 years**			
1	C25	Malignant neoplasm of pancreas	1347 (7.1)
2	C15	Malignant neoplasm of oesophagus	1201 (6.3)
3	C34	Malignant neoplasm of unspecified part of bronchus or lung	1199 (6.3)
4	C18	Malignant neoplasm of colon	1143 (6.0)
5	C22	Intrahepatic bile duct carcinoma	673 (3. 5)
6	J44	Chronic obstructive pulmonary disease	585 (3.1)
7	C16	Malignant neoplasm of stomach	577 (3.0)
8	C20	Malignant neoplasm of rectum	461 (2.4)
9	I21	Acute myocardial infarction	455 (2.4)
10	I25	Chronic ischaemic heart disease	454 (2.4)
All deaths			18 996
**Age ≥ 75 years**			
1	C18	Malignant neoplasm of colon	1472 (5.3)
2	I25	Chronic ischaemic heart disease	1059 (3.8)
3	C25	Malignant neoplasm of pancreas	1052 (3.8)
4	I21	Acute myocardial infarction	1030 (3.7)
5	J18	Pneumonia	940 (3.4)
6	C34	Malignant neoplasm of unspecified part of bronchus or lung	938 (3.4)
7	C15	Malignant neoplasm of oesophagus	923 (3.3)
8	J44	Chronic obstructive pulmonary disease	905 (3.3)
9	C61	Malignant neoplasm of prostate	628 (2.3)
10	F03	Unspecified dementia	626 (2.3)
All deaths			27 808

Values in parentheses are percentages.

The causes of death after EGS admission also varied with co-morbidity. In those without previous co-morbidity, the most common causes of death were myocardial infarction, followed by malignancy of the lung and then pneumonia. In those with moderate co-morbidity (CCI score 1–4), the most common causes of death were oesophageal cancer, COPD, and pancreatic cancer. In patients with high levels of co-morbidity (CCI score over 4), malignancy of the colon, pancreas and bronchus were the most common diagnoses at death (*Table 4*).

**Table 4:** Most common cause of death by co-morbidity status of patients who died within 1 year of emergency general surgery admission

Rank	ICD-10	Description	*n*
**No co-morbidity (CCI score 0)**			
1	I21	Acute myocardial infarction	275 (4.8)
2	C34	Malignant neoplasm of bronchus and lung	205 (3.6)
3	J18	Pneumonia, unspecified organism	192 (3.4)
4	I25	Chronic ischaemic heart disease	185 (3.2)
5	C25	Malignant neoplasm of pancreas	180 (3.2)
6	K85	Acute pancreatitis	160 (2.8)
7	K56	Paralytic ileus and intestinal obstruction without hernia	118 (2.1)
8	R68	Other general symptoms and signs (including hypothermia)	112 (2.0)
9	C80	Malignant neoplasm without specification of site	110 (1.9)
10	X42	Accidental poisoning by and exposure to narcotics and psychodysleptics	109 (1.9)
All deaths			5698
**Moderate co-morbidity (CCI score 1–4)**			
1	C15	Malignant neoplasm of oesophagus	1247 (4.8)
2	J44	Chronic obstructive pulmonary disease	1149 (4.4)
3	C25	Malignant neoplasm of pancreas	1110 (4.2)
4	I25	Chronic ischaemic heart disease	989 (3.8)
5	C34	Malignant neoplasm of bronchus and lung	942 (3.6)
6	I21	Acute myocardial infarction	908 (3.5)
7	J18	Pneumonia, unspecified organism	794 (3.0)
8	C18	Malignant neoplasm of colon	771 (2.9)
9	C22	Malignant neoplasm of liver and bile ducts	631 (2.4)
10	C16	Malignant neoplasm of stomach	547 (2.1)
All deaths			26 168
**Severe co-morbidity (CCI score > 4)**			
1	C18	Malignant neoplasm of colon	2020 (10.0)
2	C25	Malignant neoplasm of pancreas	1342 (6.6)
3	C34	Malignant neoplasm of bronchus and lung	1147 (5.7)
4	C15	Malignant neoplasm of oesophagus	1063 (5.3)
5	C50	Malignant neoplasm of breast	830 (4.1)
6	C19	Malignant neoplasm of rectosigmoid junction	695 (3.4)
7	C80	Malignant neoplasm without specification of site	693 (3.4)
8	C16	Malignant neoplasm of stomach	691 (3.4)
9	C61	Malignant neoplasm of prostate	682 (3.4)
10	C22	Malignant neoplasm of liver and bile ducts	626 (3.1)
All deaths			20 228

Values in parentheses are percentages. CCI, Charlson Co-morbidity Index.

**Fig. 1:**
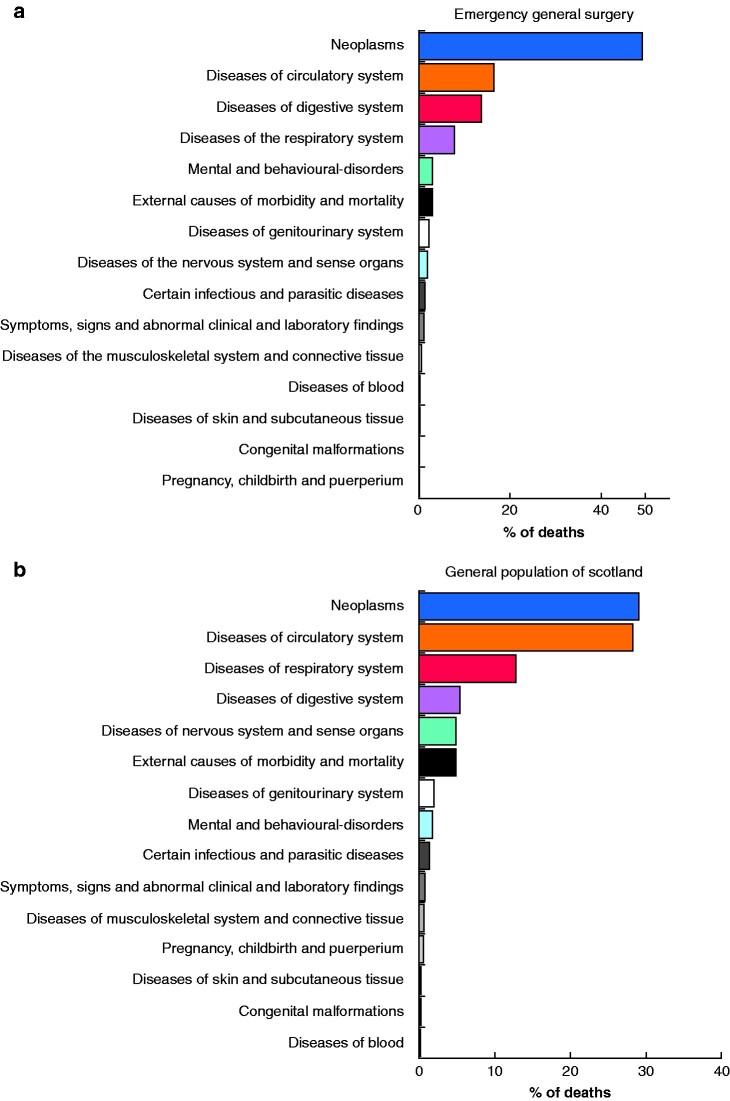
Causes of death in the emergency general surgery service and general population of Scotland over 10 years **a** Emergency general surgery and **b** general population of Scotland.

The rank order between patients treated in the EGS service and all deaths in Scotland over the same time period is shown in *Fig. 1*. Cancer represented a greater proportion of deaths in the EGS group (48 per cent) than in the Scottish population as a whole. Rates of death related to gastrointestinal pathologies were also higher in the EGS population (14 *versus* 5.8 per cent), whereas cardiovascular and respiratory diseases were less common in these patients.

### Discharge diagnoses

*Tables 1*, *2* and *Table S1* show the most common discharge diagnoses overall and by operative status. Non-specific symptoms and signs (ICD-10 R codes) represented the most common primary discharge diagnosis in 7.5 per cent of patients, followed by intestinal obstruction without hernia, colonic cancer, constipation, pancreatic cancer, and oesophageal cancer. Overall, malignancies accounted for 26 per cent of discharge diagnoses. Abdominal pain was the most common cause of admission in the non-operative cohort, followed by constipation, paralytic ileus and intestinal obstruction without hernia, colonic cancer then pancreatic cancer. Colonic cancer was the most common admission diagnosis for the operative cohort, followed by paralytic ileus and intestinal obstruction without hernia, oesophageal cancer, and pancreatic cancer.

### Association between diagnosis at discharge and cause of death

**Fig. 2:**
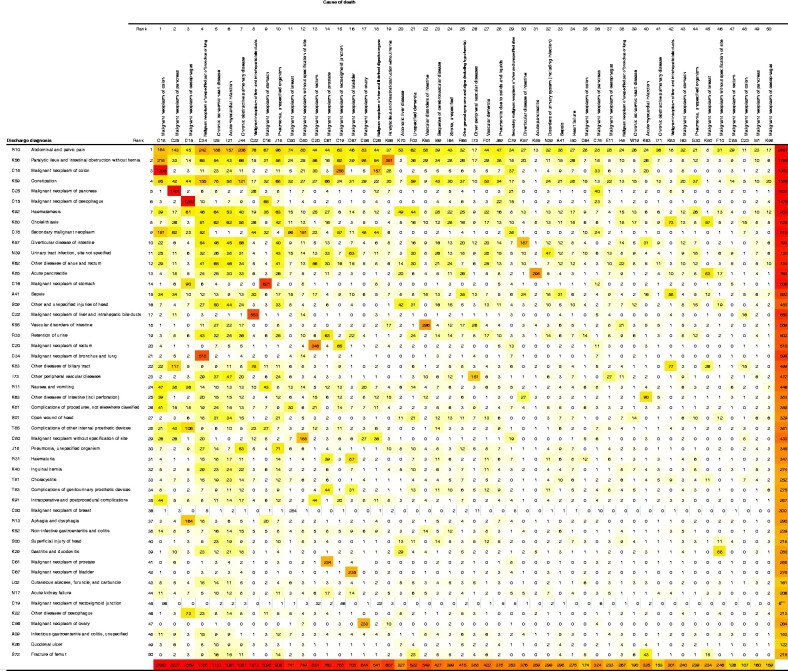
Association between diagnosis at discharge and cause of death Colour-coded matrix showing the relationship between the 50 most common primary discharge discharge diagnoses and causes of death. The number in each cell represents the number of patients with a given combination of discharge diagnosis and cause of death. The shading of the cells, from white to yellow to red, reflects the frequency. The colour scale has been set to accentuate differences in the lower range of the distribution.

*Fig. 2* shows the association between diagnosis at discharge (ranked in rows) and cause of death (ranked in columns). Given that both discharge diagnosis and causes of death are ranked, the overall trend is for numbers to decrease from the top left to bottom right. A comparison of rank order of cause of death and EGS discharge diagnosis in operative and non-operative cohorts is shown in *Figs S1* and *S2* respectively.

Cause of death was the same as the discharge diagnosis (same ICD-10 code) in 56.5 per cent of patients. Among patients who died from malignant diseases (ICD-10 C codes), the odds of the same malignancy having been known at the time of discharge were 3.10 (95 per cent c.i. 2.99 to 3.22). For patients who died from colonic cancer, the odds of having been discharged with this diagnosis in the past year were 2.42 (2.24 to 2.61); respective values were 3.69 (3.39 to 4.02) for pancreatic cancer, and 4.43 (4.03 to 4.85) for oesophageal cancer.

A symptom-based discharge diagnosis (R10, abdominal and pelvic pain; R11, nausea and vomiting; R13, aphagia and dysphagia) was associated with a wide range of causes of death. However, the odds of subsequent death from malignancy were 0.77 (0.72 to 0.82). Similarly, discharge diagnoses such as sepsis (A41), head injury (S00, S01, and S09), pneumonia (J18), superficial abscesses (L02), and infectious gastroenteritis and colitis (A0) were less obviously associated with specific causes of death.

## Discussion

The present study demonstrated that nearly half of patients who died within a year of discharge following an EGS admission succumbed to neoplastic disease. The proportion of deaths caused by malignancies in patients treated in the EGS sevice was 17 per cent higher than that for the general population of Scotland, where cancer is the cause of death in 31 per cent^20^. Other causes of death in the study cohort also differed from those in the general population. Diseases of the circulatory system were the second most common cause of death in both groups, but accounted for nearly one-third of deaths in the population as whole and only 16 per cent among the EGS cohort^20^. This is likely to reflect the management of most cardiovascular pathologies by physicians rather than emergency general surgeons. Most of the findings were largely independent of whether patients underwent operation or not.

Previous publications^21–24^ have demonstrated the importance of the cancer workload associated with EGS, but most have focused on disease-specific or operation-dependent associations. The present findings highlight the importance of cancer care in this patient population. Although interactions between the specialties of oncology, palliative care, and general surgery are well established in the elective sphere, malignancies presenting as an emergency are more likely to be at more advanced stages^25^^,^^26^, and less likely to have been discussed in a multidisciplinary format before operation^27^. Patients presenting to EGS services with malignancy may not have attended primary-care clinicians before admission^28^. Optimizing links between the EGS service, multidisciplinary team, and community care seems important.

The present findings also confirm that patients discharged with non-specific diagnoses, such as abdominal and pelvic pain, nausea and vomiting, or aphagia and dysphagia often turn out to have underlying malignancies as the subsequent cause of death^29–31^. Patients with recurring or persistent symptoms warrant further investigations. This might limit unplanned reattendances^32^ and further EGS admissions.

As expected, there were differences in causes of death between age cohorts. Self-harm, and drug and alcohol abuse were the most frequent causes of death in the younger cohort (aged 16–34 years). However, the absolute number of fatalities in this age group was very small, and therefore did not contribute markedly to the overall analysis. Increasing cohort age was associated with an increasing mortality rate. Previous work showed that the 1-year postdischarge mortality rate for patients aged over 75 years was very high at 35.6 per cent^3^. In the present analysis, malignancies, dementia, and pathologies of the lung and heart all became more prevalent in the elderly. Frailty^33^^,^^34^, and co-morbidities^35^ are known to increase mortality rates in general surgery. The present work confirmed this finding, suggesting that the elderly patients treated in EGS services represent a high-risk group who may benefit from medical optimization, with greater input from specialists in geriatric medicine^34^^,^^36^.

The present study has limitations. Although this type of study has a risk of coding errors^17^, the ISD has professional coders who work to strict standards. Consistency is monitored, and the quality of Scottish health data is thought to be high^37^^,^^38^. The accuracy of death certificates, in contrast, may be more variable, as these certificates are completed by clinicians. Although the findings may be specific to Scotland, they may still be broadly generalizable to other healthcare settings, where an increasing proportion of the population is elderly.

This study has identified that medium-term mortality following EGS admission in Scotland, regardless of whether patients undergo operation or not, is largely driven by cancer diagnoses. Around half of these diagnoses will be known at the time of discharge from inpatient care. Service integration in the hospital and community should be optimized, and surgeons providing EGS should ensure adequate follow-up, particularly for patients without a clear diagnosis on discharge because of the risk of undiagnosed malignancy.

## Funding

This study was funded by the NHS Highland Endowments fund.

*Disclosure.* The authors declare no conflict of interest.

## Supplementary material

Supplementary material is available at *BJS* online.

## Supplementary Material

zrab021_Supplementary_Data
