# Supplementary material for: Causes of death after emergency general surgical admission: population cohort study of mortality
Source: BJS Open. 2021 Apr 21;5(2):zrab021. doi: 10.1093/bjsopen/zrab021 (PMC8058150; doi:10.1093/bjsopen/zrab021)
Supplement: zrab021_Supplementary_Data [file zrab021_supplementary_data.zip › Fig. S1.pdf]

			Cause of death																									
		Rank	1	2	3	4	5	6	7	8	9	10	11	12	13	14	15	16	17	18	19	20	21	22	23	24	25	
			Malignant neoplasm of esophagus	Malignant neoplasm of colon	Malignant neoplasm of pancreas	Malignant neoplasm of unspecified part of bronchus or lung	Malignant neoplasm of stomach	Malignant neoplasm of liver and intrahepatic bile ducts	Acute myocardial infarction	Chronic ischemic heart disease	Chronic obstructive pulmonary disease	Malignant neoplasm without specification of site	Malignant neoplasm of rectum	Malignant neoplasm of rectosigmoid junction	Pneumonia, unspecified organism	Paralytic ileus and intestinal obstruction without hernia	Vascular disorders of intestine	Malignant neoplasm of ovary	Malignant neoplasm of other and ill-defined digestive organs	Malignant neoplasm of prostate	Malignant neoplasm of breast	Malignant neoplasm of bladder	Diverticular disease of intestine	Alcoholic liver disease	Stroke, unspecified	Pneumonitis due to solids and liquids	Other general symptoms and signs (including hypothermia)	
Discharge diagnosis		Rank	C15	C18	C25	C34	C16	C22	I21	I25	J44	C80	C20	C19	J18	K56	K55	C56	C26	C61	C50	C67	K57	K70	I64	J69	R68	
C18	Malignant neoplasm of colon	1	3	526	1	4	2	1	15	12	6	9	11	115	5	9	2	2	81	1	2	0	1	0	4	5	2	819
K56	Paralytic ileus and intestinal obstruction without her	2	5	78	14	28	12	7	26	26	30	11	20	17	30	165	19	23	17	5	2	19	10	1	16	15	8	604
C15	Malignant neoplasm of oesophagus	3	632	1	1	6	31	0	6	0	9	2	0	0	4	0	0	0	1	1	0	0	0	0	2	10	2	708
C25	Malignant neoplasm of pancreas	4	1	1	493	2	1	12	0	1	2	2	0	0	1	3	0	0	5	1	0	1	0	0	0	0	0	526
C78	Secondary malignant neoplasm	5	7	50	28	21	14	18	0	0	1	68	5	17	2	2	0	21	20	1	34	4	0	0	1	1	0	315
C16	Malignant neoplasm of stomach	6	41	1	1	1	267	0	0	2	11	1	1	0	0	1	0	0	3	0	0	1	0	0	2	1	0	334
K57	Diverticular disease of intestine	7	0	5	3	24	0	2	14	19	16	5	1	1	20	2	2	1	2	7	4	3	87	1	6	3	7	235
K80	Cholelithiasis	8	0	4	9	8	1	11	15	12	3	2	0	1	16	0	2	1	0	2	4	1	1	0	10	7	3	113
K55	Vascular disorders of intestine	9	0	0	0	2	0	0	16	7	16	1	2	1	4	12	140	0	2	0	3	2	0	1	3	2	9	223
R33	Retention of urine	10	2	1	5	15	3	2	7	13	2	6	4	1	12	1	1	3	0	32	0	8	0	2	8	6	5	139
T85	Complications of other internal prosthetic devices	11	65	12	23	7	15	9	7	2	3	0	5	6	4	1	0	2	2	1	0	1	0	0	33	4	1	203
C20	Malignant neoplasm of rectum	12	0	18	1	0	1	0	5	6	12	4	135	30	1	2	0	0	7	1	0	1	0	0	2	4	1	231
K92	Hematemesis	13	12	4	4	6	4	1	15	16	11	2	3	0	11	0	1	0	1	3	3	4	1	19	2	4	7	134
K62	Other diseases of anus and rectum	14	1	6	2	6	1	0	7	12	1	3	9	5	10	3	1	0	1	7	4	4	2	0	7	0	2	94
C22	Malignant neoplasm of liver and hepatic bile ducts	15	0	0	3	1	1	185	1	0	3	5	0	0	1	1	0	0	1	0	1	0	0	0	1	0	0	204
K63	Other diseases of intestine (incl perforation)	16	1	13	0	7	1	2	10	6	11	0	5	7	6	10	7	2	2	2	2	0	14	0	0	2	4	114
R10	Abdominal and pelvic pain	17	4	15	6	8	7	3	7	15	3	8	1	4	4	2	1	6	0	2	4	1	1	2	5	5	0	114
L02	Cutaneous abscess, furuncle and carbuncle	18	4	4	4	13	2	0	10	8	10	2	1	3	2	0	3	0	1	2	5	4	1	4	3	3	2	91
K40	Inguinal hernia	19	2	2	1	15	2	1	12	16	6	2	1	0	7	1	2	1	0	2	2	4	1	3	2	2	3	90
K26	Duodenal ulcer	20	0	0	3	5	2	1	5	9	7	2	1	0	2	0	0	1	0	0	1	1	1	3	1	3	4	52
K22	Other diseases of esophagus	21	49	1	1	13	7	0	10	6	1	65	1	0	4	0	0	0	0	3	2	1	0	6	2	3	4	179
C80	Malignant neoplasm without specification of site	22	0	10	9	8	5	4	0	0	9	2	0	2	0	1	0	13	19	1	2	3	0	0	1	1	1	91
K59	Constipation	23	2	11	2	9	4	1	1	10	0	5	3	3	9	6	0	0	0	6	2	3	1	0	4	2	3	87
K83	Other diseases of biliary tract	24	2	6	50	2	4	23	2	3	4	1	1	1	4	0	3	0	2	0	3	1	0	0	1	1	2	116
R13	Aphagia and dysphagia	25	84	2	1	9	8	0	3	2	9	0	0	0	4	0	0	0	1	12	1	0	0	0	0	2	1	139
			917	771	665	220	395	283	194	203	186	208	210	214	163	222	184	76	168	92	81	67	121	42	116	86	71	
