# Supplementary material for: Causes of death after emergency general surgical admission: population cohort study of mortality
Source: BJS Open. 2021 Apr 21;5(2):zrab021. doi: 10.1093/bjsopen/zrab021 (PMC8058150; doi:10.1093/bjsopen/zrab021)
Supplement: zrab021_Supplementary_Data [file zrab021_supplementary_data.zip › Fig. S2.pdf]

			Cause of death																									
		Rank	1	2	3	4	5	6	7	8	9	10	11	12	13	14	15	16	17	18	19	20	21	22	23	24	25	
			Malignant neoplasm of colon	Malignant neoplasm of unspecified part of bronchus or lung	Malignant neoplasm of pancreas	Malignant neoplasm of esophagus	Chronic ischemic heart disease	Chronic obstructive pulmonary disease	Acute myocardial infarction	Malignant neoplasm of liver and intrahepatic bile ducts	Pneumonia, unspecified organism	Malignant neoplasm of breast	Malignant neoplasm of stomach	Malignant neoplasm of prostate	Malignant neoplasm of bladder	Malignant neoplasm of rectum	Malignant neoplasm without specification of site	Malignant neoplasm of rectosigmoid junction	Malignant neoplasm of ovary	Unspecified dementia	Alcoholic liver disease	Malignant neoplasm of other and ill-defined digestive organs	Sequelae of cerebrovascular disease	Other peripheral vascular diseases	Vascular dementia	Paralytic ileus and intestinal obstruction without hernia	Other general symptoms and signs (including hypothermia)	
Discharge diagnosis		Rank	C18	C34	C25	C15	I25	J44	I21	C22	J18	C50	C16	C61	C67	C20	C80	C19	C56	F03	K70	C26	I69	I73	F01	K56	R68	
R10	Abdominal and pelvic pain	1	169	234	137	41	171	195	150	73	92	70	60	42	45	43	72	59	77	60	51	44	38	17	42	35	32	2049
K59	Constipation	2	84	146	40	42	68	112	57	16	59	30	28	60	28	24	25	22	29	56	7	22	40	10	47	24	34	1110
K56	Paralytic ileus and intestinal obstruction without her	3	140	37	19	9	28	36	17	8	28	22	19	11	20	36	14	45	75	23	2	37	23	12	14	186	18	879
C18	Malignant neoplasm of colon	4	680	7	1	0	11	5	9	1	6	3	0	2	1	19	12	143	3	0	0	76	4	2	4	19	4	1012
C25	Malignant neoplasm of pancreas	5	0	4	968	1	7	0	2	14	7	1	2	3	0	0	5	1	0	0	0	7	2	0	0	4	4	1032
K92	Hematemesis	6	35	40	13	49	48	28	38	18	52	12	31	24	10	22	8	6	8	41	30	11	24	6	20	2	18	594
N39	Urinary tract infection, site not specified	7	22	30	11	5	22	30	34	4	42	15	1	28	61	9	9	7	5	27	3	11	24	8	27	6	17	458
K85	Acute pancreatitis	8	3	21	17	4	20	30	26	6	25	6	1	9	2	2	5	1	0	5	17	1	5	0	3	1	25	235
K80	Cholelithiasis	9	3	43	17	3	50	42	47	15	26	7	4	14	2	1	11	1	4	11	5	0	11	7	14	4	13	355
C15	Malignant neoplasm of oesophagus	10	2	4	1	628	1	0	1	0	3	1	34	1	1	0	2	0	0	0	0	3	3	0	3	0	4	692
K62	Other diseases of anus and rectum	11	23	35	9	2	42	23	39	5	31	3	3	23	11	47	10	11	5	20	14	7	16	5	20	3	5	412
S09	Other and unspecified injuries of head	12	7	27	4	7	50	23	43	3	33	8	3	5	4	2	4	0	0	30	40	0	16	3	13	1	4	330
K57	Diverticular disease of intestine	13	17	30	3	4	27	45	31	2	20	5	2	6	4	4	6	1	3	15	1	4	11	10	16	6	13	286
C78	Secondary malignant neoplasm	14	101	31	34	16	1	1	1	26	2	62	18	3	7	18	83	40	27	0	0	24	2	1	0	4	4	506
A41	Sepsis	15	27	10	26	10	11	12	5	22	15	13	5	4	7	15	6	8	2	5	7	4	11	12	7	7	24	275
C34	Malignant neoplasm of bronchus and lung	16	2	437	5	2	1	4	1	2	2	3	0	1	1	2	6	2	0	0	0	1	1	3	0	0	1	477
R11	Nausea and vomiting	17	44	12	35	34	9	8	13	9	7	13	40	10	3	4	13	11	19	13	6	6	6	3	6	4	2	330
C16	Malignant neoplasm of stomach	18	0	5	5	49	2	1	3	0	2	0	354	1	0	0	7	0	0	0	0	5	1	0	0	2	0	437
I73	Other peripheral vascular diseases	19	2	25	2	1	31	15	30	2	21	6	5	2	1	4	2	2	1	3	0	1	6	128	5	0	1	296
S01	Open wound of head	20	2	16	3	6	31	10	34	1	20	1	2	2	0	3	5	0	3	19	11	1	12	7	12	0	11	212
C22	Malignant neoplasm of liver and intrahepatic bile duc	21	2	2	8	0	2	0	0	368	0	1	0	1	0	0	9	1	0	1	2	2	1	0	0	0	1	401
J18	Pneumonia, unspecified organism	22	6	26	2	8	14	53	7	5	66	6	3	4	1	1	6	4	0	10	6	1	7	4	8	10	12	270
K83	Other diseases of biliary tract	23	16	7	67	3	6	8	9	55	7	23	7	1	0	2	3	6	0	6	0	1	1	2	4	0	6	240
T81	Cholecystitis	24	30	14	14	12	19	11	12	5	9	0	6	0	5	15	4	12	6	2	2	8	4	8	2	3	3	206
K55	Vascular disorders of intestine	25	1	9	1	0	20	14	6	0	4	1	0	0	0	0	2	1	12	0	1	0	2	19	4	5	8	110
			1418	1252	1442	936	692	706	615	660	579	312	628	257	214	273	329	384	279	347	205	277	271	267	271	326	264	
