# Supplementary material for: Causes of death after emergency general surgical admission: population cohort study of mortality
Source: BJS Open. 2021 Apr 21;5(2):zrab021. doi: 10.1093/bjsopen/zrab021 (PMC8058150; doi:10.1093/bjsopen/zrab021)
Supplement: zrab021_Supplementary_Data [file zrab021_supplementary_data.zip › Table S1 and figure legends.docx]

**Table S1** Most common discharge diagnoses and causes of death for the 52 094 patients who died within one year of EGS admission. Percentages are percentages of all deaths (not just those listed in this table).

| **Causes of death** | | | | | **Discharge diagnoses** | | | | |
| --- | --- | --- | --- | --- | --- | --- | --- | --- | --- |
| **Rank** | **ICD-10** | **Description** | **n** | **(%)** | **Rank** | **ICD-10** | **Description** | **n** | **(%)** |
| **1** | C18 | Malignant neoplasm of colon | 2 883 | (5.53) | **1** | R10 | Abdominal and pelvic pain | 3 913 | (7.51) |
| **2** | C25 | Malignant neoplasm of pancreas | 2 632 | (5.05) | **2** | K56 | Paralytic ileus and intestinal obstruction without hernia | 2 241 | (4.30) |
| **3** | C15 | Malignant neoplasm of esophagus | 2 349 | (4.51) | **3** | C18 | Malignant neoplasm of colon | 2 108 | (4.05) |
| **4** | C34 | Malignant neoplasm of unspecified part of bronchus or lung | 2 294 | (4.40) | **4** | K59 | Constipation | 2 096 | (4.02) |
| **5** | I25 | Chronic ischemic heart disease | 1 617 | (3.10) | **5** | C25 | Malignant neoplasm of pancreas | 1 732 | (3.32) |
| **6** | I21 | Acute myocardial infarction | 1 542 | (2.96) | **6** | C15 | Malignant neoplasm of esophagus | 1 540 | (2.96) |
| **7** | J44 | Chronic obstructive pulmonary disease | 1 528 | (2.93) | **7** | K92 | Hematemesis | 1 354 | (2.60) |
| **8** | C22 | Malignant neoplasm of liver and intrahepatic bile ducts | 1 344 | (2.58) | **8** | K80 | Cholelithiasis | 1 160 | (2.23) |
| **9** | C16 | Malignant neoplasm of stomach | 1 279 | (2.46) | **9** | C78 | Secondary malignant neoplasm of respiratory and digestive organs | 1 064 | (2.04) |
| **10** | J18 | Pneumonia, unspecified organism | 1 215 | (2.33) | **10** | K57 | Diverticular disease of intestine | 1 033 | (1.98) |
| **11** | C50 | Malignant neoplasm of breast | 1 014 | (1.95) | **11** | N39 | Urinary tract infection, site not specified | 992 | (1.90) |
| **12** | C80 | Malignant neoplasm without specification of site | 1 009 | (1.94) | **12** | K62 | Other diseases of anus and rectum | 984 | (1.89) |
| **13** | C20 | Malignant neoplasm of rectum | 994 | (1.91) | **13** | K85 | Acute pancreatitis | 963 | (1.85) |
| **14** | C61 | Malignant neoplasm of prostate | 978 | (1.88) | **14** | C16 | Malignant neoplasm of stomach | 847 | (1.63) |
| **15** | C19 | Malignant neoplasm of rectosigmoid junction | 904 | (1.74) | **15** | A41 | Sepsis | 809 | (1.55) |
| **16** | C67 | Malignant neoplasm of bladder | 902 | (1.73) | **16** | S09 | Other and unspecified injuries of head | 731 | (1.40) |
| **17** | C56 | Malignant neoplasm of ovary | 794 | (1.52) | **17** | C22 | Malignant neoplasm of liver and intrahepatic bile ducts | 694 | (1.33) |
| **18** | C26 | Malignant neoplasm of other and ill-defined digestive organs | 710 | (1.36) | **18** | K55 | Vascular disorders of intestine | 685 | (1.31) |
| **19** | K56 | Paralytic ileus and intestinal obstruction without hernia | 696 | (1.34) | **19** | R33 | Retention of urine | 629 | (1.21) |
| **20** | K70 | Alcoholic liver disease | 692 | (1.33) | **20** | C20 | Malignant neoplasm of rectum | 628 | (1.21) |
| **21** | F03 | Unspecified dementia | 667 | (1.28) | **21** | C34 | Malignant neoplasm of bronchus and lung | 619 | (1.19) |
| **22** | K55 | Vascular disorders of intestine | 640 | (1.23) | **22** | K83 | Other diseases of biliary tract | 598 | (1.15) |
| **23** | I69 | Sequelae of cerebrovascular disease | 576 | (1.11) | **23** | I73 | Other peripheral vascular diseases | 590 | (1.13) |
| **24** | I64 | Stroke, unspecified | 571 | (1.10) | **24** | R11 | Nausea and vomiting | 550 | (1.06) |
| **25** | R68 | Other general symptoms and signs (including hypothermia) | 561 | (1.08) | **25** | K63 | Other diseases of intestine (incl perforation) | 520 | (1.00) |
| **26** | I73 | Other peripheral vascular diseases | 553 | (1.06) | **26** | S01 | Open wound of head | 515 | (0.99) |
| **27** | F01 | Vascular dementia | 544 | (1.04) | **27** | T81 | Complications of procedures, not elsewhere classified | 515 | (0.99) |
| **28** | J69 | Pneumonitis due to solids and liquids | 637 | (1.22) | **28** | T85 | Complications of other internal prosthetic devices, implants and grafts | 502 | (0.96) |
| **29** | C79 | Secondary malignant neoplasm of other and unspecified sites | 459 | (0.88) | **29** | C80 | Malignant neoplasm without specification of site | 496 | (0.95) |
| **30** | K57 | Diverticular disease of intestine | 453 | (0.87) | **30** | J18 | Pneumonia, unspecified organism | 469 | (0.90) |
| **31** | K85 | Acute pancreatitis | 422 | (0.81) | **31** | R31 | Haematuria | 441 | (0.85) |
| **32** | N39 | Disorders of urinary system (including infection) | 420 | (0.81) | **32** | K40 | Inguinal hernia | 412 | (0.79) |
| **33** | A41 | Sepsis | 416 | (0.80) | **33** | K81 | Cholecystitis | 398 | (0.76) |
| **34** | I50 | Heart failure | 406 | (0.78) | **34** | T83 | Complications of genitourinary prosthetic devices, implants and grafts | 376 | (0.72) |
| **35** | C64 | Malignant neoplasm of kidney, except renal pelvis | 399 | (0.77) | **35** | K91 | Intraoperative and postprocedural complications and disorders of […] | 371 | (0.71) |
| **36** | C78 | Secondary malignant neoplasm of respiratory and digestive organs | 394 | (0.76) | **36** | C50 | Malignant neoplasm of breast | 361 | (0.69) |
| **37** | E11 | Type 2 diabetes mellitus | 378 | (0.73) | **37** | R13 | Aphagia and dysphagia | 338 | (0.65) |
| **38** | I48 | Atrial fibrillation and flutter | 375 | (0.72) | **38** | K52 | Other and unspecified noninfective gastroenteritis and colitis | 335 | (0.64) |
| **39** | W19 | Unspecified fall | 367 | (0.70) | **39** | S00 | Superficial injury of head | 322 | (0.62) |
| **40** | K63 | Other diseases of intestine (including perforation) | 364 | (0.70) | **40** | C61 | Malignant neoplasm of prostate | 312 | (0.60) |
| **41** | I71 | Aortic aneurysm and dissection | 357 | (0.69) | **41** | K29 | Gastritis and duodenitis | 312 | (0.60) |
| **42** | K83 | Diseases of biliary tract (including cholangitis) | 349 | (0.67) | **42** | C67 | Malignant neoplasm of bladder | 308 | (0.59) |
| **43** | G30 | Alzheimer's disease | 343 | (0.66) | **43** | L02 | Cutaneous abscess, furuncle and carbuncle | 305 | (0.59) |
| **44** | I63 | Cerebral infarction | 343 | (0.66) | **44** | N17 | Acute kidney failure | 297 | (0.57) |
| **45** | K80 | Cholelithiasis (including cholecystitis) | 252 | (0.48) | **45** | C19 | Malignant neoplasm of rectosigmoid junction | 291 | (0.56) |
| **46** | F10 | Alcohol related disorders | 249 | (0.48) | **46** | K22 | Other diseases of oesophagus | 289 | (0.55) |
| **47** | C85 | Specified and unspecified types of non-Hodgkin lymphoma | 247 | (0.47) | **47** | A09 | Infectious gastroenteritis and colitis, unspecified | 287 | (0.55) |
| **48** | C23 | Malignant neoplasm of gallbladder | 238 | (0.46) | **48** | C45 | Mesothelioma | 287 | (0.55) |
| **49** | I51 | Heart disease, unspecified | 227 | (0.44) | **49** | K26 | Duodenal ulcer | 275 | (0.53) |
| **50** | J98 | Other Respiratory Disorders | 217 | (0.42) | **50** | I71 | Aortic aneurysm and dissection | 283 | (0.54) |

**Fig. S1 Association between diagnosis at discharge and cause of death in operative cohort**

**Fig. S2 Association between diagnosis at discharge and cause of death in non-operative cohort**
